# Comment to: II Brazilian Consensus on the use of human immunoglobulin in patients with primary immunodeficiencies. einstein (São Paulo). 2017;15(1):1-16

**DOI:** 10.1590/S1679-45082017CE4250

**Published:** 2017

**Authors:** Ekaterini Simões Goudouris, Almerinda Maria do Rego Silva, Aluce Loureiro Ouricuri, Anete Sevciovic Grumach, Antonio Condino-Neto, Beatriz Tavares Costa-Carvalho, Carolina Cardoso de Mello Prando, Cristina Maria Kokron, Dewton de Moraes Vasconcelos, Fabíola Scancetti Tavares, Gesmar Rodrigues Silva Segundo, Irma Cecília Douglas Paes Barreto, Mayra de Barros Dorna, Myrthes Anna Maragna Toledo Barros, Wilma Carvalho Neves Forte

**Affiliations:** Universidade Federal do Rio de Janeiro, Rio de Janeiro, RJ, Brazil; Universidade Federal de Pernambuco, Recife, PE, Brazil; Hospital Servidores do Estado do Rio de Janeiro, Rio de Janeiro, RJ, Brazil; Faculdade de Medicina do ABC, Santo André, SP, Brazil; Universidade de São Paulo, São Paulo, SP, Brazil; Universidade Federal de São Paulo, São Paulo, SP, Brazil; Hospital Pequeno Príncipe, Curitiba, PR, Brazil; Hospital das Clínicas, Faculdade de Medicina, Universidade de São Paulo, São Paulo, SP, Brazil; Hospital das Clínicas, Faculdade de Medicina, Universidade de São Paulo, São Paulo, SP, Brazil; Hospital de Base do Distrito Federal, Brasília, DF, Brazil; Universidade Federal de Uberlândia, Uberlândia, MG, Brazil; Centro Universitário do Estado do Pará, Belém, PA, Brazil; Faculdade de Medicina, Universidade de São Paulo, São Paulo, SP, Brazil; Faculdade de Medicina, Universidade de São Paulo, São Paulo, SP, Brazil; Faculdade de Ciências Médicas, Santa Casa de São Paulo, São Paulo, SP, Brazil

Dear editor,

We would like to update the readership of **einstein** (São Paulo) on the information given in page 6, regarding hyaluronidase facilitated subcutaneous immunoglobulin, in the article entitled II Brazilian Consensus on the use of human immunoglobulin in patients with primary immunodeficiencies published in this journal in volume 15 issue 1, 2017.^(^ ^1^ ^)^

When the article was written information available was that hyaluronidase facilitated subcutaneous immunoglobulin was not approved to be used by children and pregnant women, even in countries that the product is commercially available. However, in Europe the product is approved to be used by children of any age since July 2016.^(^ ^2^ ^–^ ^4^ ^)^ The hyaluronidase facilitated subcutaneous immunoglobulin infusion use in pregnant women, however, is still under investigation^(^ ^4^ ^)^

To correct this information is relevant because it guarantees that our article can provide the most accurate information as possible. In addition, patients should learn about the availability of this new therapeutic resource until this medication becomes approved in our country.
